# Distinguishing functional and structural MRI abnormalities between bipolar and unipolar depression

**DOI:** 10.3389/fpsyt.2023.1343195

**Published:** 2023-12-19

**Authors:** Shiqing Huang, Xiaoxia Wen, Zhiling Liu, Cuiyun Li, Yuqiu He, Jiaquan Liang, Wei Huang

**Affiliations:** Department of Psychiatry, The Third People’s Hospital of Foshan, Foshan, Guangdong, China

**Keywords:** bipolar disorder, major depressive disorder, fractional amplitude of low frequency fluctuation, regional homogeneity, magnetic resonance imaging – high field

## Abstract

**Background:**

This study aims to investigate the underlying characteristics of spontaneous brain activity by analyzing the volumes of the hippocampus and parahippocampal gyrus, as well as the fractional amplitude of low-frequency fluctuation (fALFF) and regional homogeneity (ReHo), in order to differentiate between bipolar disorder (BD) and unipolar depressive disorder.

**Methods:**

A total of 46 healthy controls, 58 patients with major depressive disorder (MDD), and 61 patients with BD participated in the study and underwent resting-state functional magnetic resonance imaging (rs-fMRI) scans. The researchers calculated the differences in volume, fALFF, and ReHo values among the three groups. Additionally, they conducted correlation analyses to examine the relationships between clinical variables and the aforementioned brain measures.

**Results:**

The results showed that the BD group exhibited increased fALFF in the hippocampus compared to the healthy control (HC) and MDD groups. Furthermore, the ReHo values in the hippocampus and parahippocampal gyrus were significantly higher in the BD group compared to the HC group. The findings from the person correlation analysis indicated a positive relationship between ReHo values in the hippocampus and both HAMD and HAMA scores. Moreover, there was no correlation between the volumes, fALFF, and ReHo values in the hippocampus and parahippocampal gyrus, and cognitive function levels (RBANS).

**Conclusion:**

Taken together, these aberrant patterns of intrinsic brain activity in the hippocampus and parahippocampal gyrus may serve as quantitative indicators for distinguishing between BD and unipolar depression.

## Introduction

Bipolar disorder (BD) is a complex psychiatric condition characterized by alternating episodes of depression and manic or hypomanic states (1), often accompanied by cognitive impairments and impulsive behaviors related to emotions (2). The challenge lies in differentiating BD from major depressive disorder (MDD) (3), as symptoms of depression in BD can often be mistaken for MDD. Unfortunately, misdiagnosis is common, with many BD patients being incorrectly identified as having MDD for extended periods of time (4). This can have serious consequences, including worsened manic symptoms, decreased quality of life, and an increased risk of suicide (5). To ensure accurate diagnosis, clinicians need to be aware of the comorbidities associated with BD and develop means to distinguish it from other disorders. Differentiating between bipolar and unipolar depression based solely on clinical observations can be challenging, leading researchers to explore neural markers through neuroimaging in order to distinguish between the two (6). Therefore, it is necessary to identify biomarkers associated with bipolar depression and develop clinically applicable diagnostic tools to shed light on its potential pathogenesis (7).

The regulation of emotions is closely linked to the hippocampus and parahippocampal gyrus (8), and these brain regions are also involved in cognitive functioning (9). Some studies have revealed abnormal brain activity in the hippocampus among BD patients and those at high risk of developing the disorder (10, 11). The hippocampus, a key component of the limbic system, is known to be involved in various cognitive functions, such as memory formation, consolidation, and retrieval (12). Alterations in hippocampal structure and function have been consistently observed in both depression and bipolar disorder, suggesting that these disorders may have shared underlying pathophysiology (13). The parahippocampal gyrus, which borders the hippocampus, is involved in sensory processing, attention, and spatial navigation. It also plays a role in the regulation of emotions and has been reported to exhibit changes in patients with mood disorders (14). Furthermore, a meta-analysis has reported functional and/or structural abnormalities in both the hippocampus and parahippocampal gyrus, suggesting that these regions are vulnerable in individuals with BD and may be responsible for early impairments in declarative memory (15). Therefore, investigating the hippocampus and parahippocampal gyrus in bipolar and unipolar depression may provide valuable insights into the underlying neural mechanisms associated with these conditions. By examining these regions, we can potentially identify biomarkers or diagnostic indicators that distinguish between these two major mood disorders, as well as understand the neural substrates of cognitive and affective symptoms. Thus, the choice of these specific brain regions for study is crucial in the pursuit of developing more targeted and effective treatments for bipolar and unipolar depression. Further depth in explaining the selection of the hippocampus and parahippocampal gyrus in research involving these disorders will enhance the understanding of their role in the pathophysiology and treatment of mood disorders.

In the last decades, functional magnetic resonance imaging (fMRI) has allowed to explore brain function both during the performance of a task and at rest. Particularly, resting-state fMRI has been widely used to analyze the differences in spontaneous brain activity and functional connectivity of various brain regions through various measures, including fractional Amplitude of Low-Frequency Fluctuations (fALFF) and Regional Homogeneity (ReHo) (16). The fALFF is a method for quantifying spontaneous brain activity by measuring the intensity fluctuation of fMRI signals with specific frequencies in a given region of interest (17). It reflects the synchronization of neuronal oscillations within a region and has been used to investigate various neurological and psychiatric disorders (18). ReHo is another fMRI-based method for evaluating functional coherence within regions of interest. It measures the similarity of fMRI signal time series within a given region by calculating the correlation coefficient of fMRI signal fluctuations over specified periods of time (19). ReHo has been used to investigate cognitive processes, emotional regulation, and neurological disorders (20).

Given these findings, this study aims to investigate the diagnosis of bipolar and unipolar depression by integrating results from psychological assessments, fMRI scans, and cognitive evaluations. We hypothesize that functional abnormalities and cognitive differences in the hippocampus and parahippocampal gyrus can serve as distinguishing features between unipolar depression and bipolar depression. Therefore, we propose to examine data from the Hamilton Depression Rating Scale (HAMD), Hamilton Anxiety Rating Scale (HAMA), fMRI scans, Repeatable Battery for the Assessment of Neuropsychological Status (RBANS) scores, and the ten cognitive domains of the RBANS scale. The aim of this study is to offer valuable perspectives for future studies on the diagnosis of bipolar depression and unipolar depressive disorders, contributing to a more comprehensive understanding of these conditions and informing more effective treatments.

## Method

### Participants

Participants (both MDD and BD groups) were recruited from the Third People’s Hospital of Foshan. Patients were diagnosed according to the Structure interview of Diagnostic and Statistical Manual-5th edition (DSM-5). Notably, all BD patients were in a depressive phase (We used the 24-item HAMD for assessing depressive symptoms) (3). Healthy control (HC) participants were selected from local communities, matching the MDD and BD participants in terms of age, gender, education, and other relevant factors. Ethics Number: FSSY-LS202201.

### Inclusion and exclusion criteria

Inclusion criteria for participants included being of Han nationality, right-handedness, having a first-episode drug-naïve mental illness, and no family history or underlying diseases. Diagnosis criteria for MDD or BD were based on the DSM-5. Exclusion criteria comprised contraindications to fMRI acquisition, the presence of brain organic or other physical diseases, substance abuse (including drugs and alcohol), traumatic brain injuries, and nervous system diseases, among others.

### Scale assessment

Participants underwent assessment using the Hamilton Depression Rating Scale (HAMD), Hamilton Anxiety Rating Scale (HAMA), and RBANS scores. The HAMD questionnaire assessed the severity of the disease, while RBANS scores aimed at evaluating cognitive function. The RBANS scale is a comprehensive neuropsychological assessment tool designed to evaluate a broad range of cognitive functions in adults (21). The scale consists of a series of standardized tests and tasks that aim to assess various cognitive domains, including attention, memory, language, executive functions, and visual–spatial abilities. These tasks are designed to be repeated and can be administered over multiple sessions to assess changes in cognitive performance over time. The scale provides quantitative scores that allow clinicians and researchers to compare an individual’s cognitive performance to established norms based on age, education, and other relevant factors (22).

### MRI acquisition

MRI acquisition was conducted using a General Electric 3 T Excite HD scanner. The scan parameters were as follows: Time repetition (TR)/Echo time (TE) = 8.6/3.3 ms, Flip angle (FA) = 9°, Field of view (FOV) = 256 mm * 256 mm, layer thickness = 1 mm, slice number = 172. For resting brain function MRI acquisition, parameters were TR/TE = 2000/30 ms, FA = 90°, FOV = 240 mm * 240 mm, layer thickness = 4 mm, number of layers = 36, and layer spacing = 1 mm.

### Fractional amplitude of Low-frequency fluctuations analysis

The fALFF analysis was conducted following a previously established methodology (23). Essentially, the energy of each frequency within the low-frequency range (0.01 Hz < *f* < 0.1 Hz) was divided by the energy of each frequency across the entire frequency range to calculate the fALFF value for each voxel. This value was then normalized by dividing it by the average amplitude of the entire brain signal to account for overall level differences in fALFF.

### Regional homogeneity analysis

ReHo analysis involved clustering twenty-seven voxels and applying the Kendall consistency coefficient (KCC) to measure the similarity between a voxel and its twenty-six neighboring voxels. The DPARSF software’s standard brain model was used to obtain KCC maps for each subject. Subsequently, the KCC value for each voxel was normalized by dividing it by the average value from the standard brain model, resulting in standardized mean ReHo maps. These maps were then smoothed.

### Data Preprocessing and processing

To ensure comprehensive assessments, all participants were requested to complete scale evaluations and fMRI data collection on the same day. Upon completion, fMRI images were visually examined to guarantee their quality and eliminate any unwanted artifacts or noise. Subsequently, the fMRI data was normalized to the MNI-152 template employing SPM8, and functional MRI data was registered to the structural fMRI using the registration tool in SPM8. To achieve higher precision, the fMRI data was resampled to a resolution of 2 mm x 2 mm x 2 mm. To further enhance the data quality, ffMRI data was smoothed using a Gaussian kernel with a full-width at half-maximum (FWHM) of 8 mm.

The data processing assistant for Resting-State fMRI (DPARSF), SPM8, and cat12 software were employed to preprocess the fMRI data. This included measuring the volume of the hippocampus and parahippocampal gyrus and analyzing the neural activity in these regions using fALFF and ReHo. Finally, the ReHo maps underwent spatial smoothening through the utilization of an 8 mm full-width at half maximum Gaussian kernel.

### Statistical analyses

Statistical analyses were conducted using SPSS 24.00. The Kolmogorov–Smirnov test assessed the probability distribution of each group, and the results of all groups showed that they all obeyed normal distribution. The significance between groups was calculated by one-way analysis of variance (ANOVA) followed by a *post hoc* test, and all values are presented as means ± standard deviation (SD). Pearson correlation analysis was used to examine the relationships between hippocampal/parahippocampal gyrus volume and functional values and clinical data. Finally, *p*-values were corrected for multiple comparisons.

## Results

### Patient characteristics

**Figure 1:**
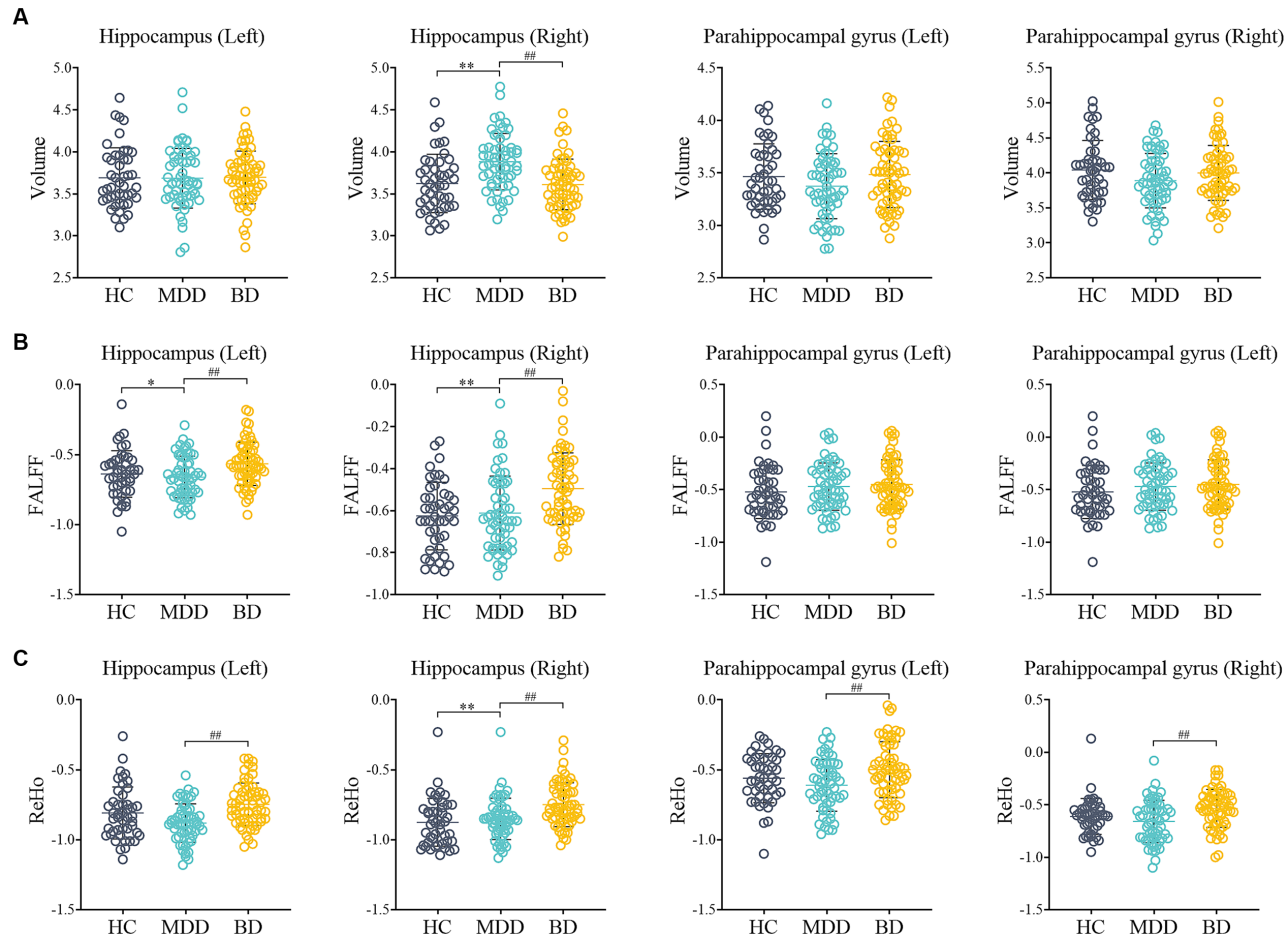
Comparison of MRI data among HC, MDD and BD. **(A)** The volume of Hippocampus (Left, Right) and Parahippocampal gyrus (Left, Right); **(B)** The fALFF of Hippocampus (Left, Right) and Parahippocampal gyrus (Left, Right); **(C)** The ReHo of Hippocampus (Left, Right) and Parahippocampal gyrus (Left, Right); HC: healthy control; MDD: major depressive disorder; BD: bipolar disorder; fALFF: fractional amplitude of low frequency fluctuation; Reho: regional homogeneity; ^*^p < 0.05, ^**^p < 0.01 compared to HC group; ^#^*p* < 0.05, ^##^*p* < 0.01 compared to MDD group.

A total of 165 participants were recruited for this study, including healthy controls (n = 46), patients with MDD (n = 58), and patients with BD (n = 61). There were no significant differences in age, gender, body mass index (BMI), and years of education among the HC, MDD, and BD groups (Table 1). However, the BD group exhibited significantly lower scores in various cognitive domains, including immediate memory (learning and story memory), attention (coding and digit span), and delayed memory (list recognition, story recall, and figure recall), compared to the HC and MDD groups. Moreover, the BD group had lower attention (digit span) scores compared to the HC group. There were no significant differences in visuospatial construction and language among the HC, MDD, and BD groups (Table 2; Figure 1).

**Table 1:** Description and comparison of Clinical Scales among Healthy Control, Major Depressive Disorder, and Bipolar Disorder Groups.

	HC	MDD	BD	Statistics
Participants	46	58	61	
Age (years)	32.20 ± 10.36	29.29 ± 12.44	30.05 ± 9.11	*F* = 0.136; *p* = 0.375
Gender (M/F)	19/27	23/35	23/38	χ2 = 0.241; *p* = 0.969
BMI	22.24 ± 3.43	22.23 ± 3.85	23.29 ± 3.76	*F* = 0.220; *p* = 0.217
Education				
Below 9 years, *n*	14	15	19	χ2 = 0.293; *p* = 0.864
9 years and above, *n*	32	43	42	
HAMD score	2.5 ± 3.67	23.53 ± 7.40^**^	14.07 ± 9.29^**##^	*F* = 102.3; P^**^<0.001, *P*^##^<0.001
HAMA score	2.02 ± 2.59	16.04 ± 5.53^**^	9.87 ± 7.43^**##^	*F* = 74.3; P^**^<0.001, *P*^##^<0.001
RBANS	190.2 ± 39.50	181.2 ± 35.58	156.8 ± 33.14^**##^	*F* = 12.84; P^**^<0.001, *P*^##^<0.001
Immediate memory (Learning)	27.63 ± 7.03	26.22 ± 6.49	21.89 ± 6.36^**##^	*F* = 11.45; P^**^<0.001, *P*^##^<0.001
Immediate memory (Story Memory)	14.41 ± 5.96	13.05 ± 5.66	9.41 ± 4.66^**##^	*F* = 12.70; P^**^<0.001, *P*^##^<0.001
Visuospatial Construction	17.76 ± 2.41	18.47 ± 2.38	17.31 ± 3.32	*F* = 2.602; *p* = 0.077
Language	18.28 ± 4.34	17.36 ± 4.70	16.52 ± 4.44	*F* = 2.00; *p* = 0.139
Attention (Digit span)	14.13 ± 2.18	13.52 ± 2.68	12.75 ± 5.54^*^	*F* = 4.069; P^*^ = 0.015
Attention (Coding)	49.80 ± 14.15	46.57 ± 13.20	40.43 ± 13.46^**#^	*F* = 6.721; P^**^ = 0.002, *P*^#^ = 0.039
Delayed memory (List Recall)	6.61 ± 3.11	5.88 ± 2.94	4.66 ± 2.53^**^	*F* = 6.523; P^**^ = 0.002
Delayed memory (List Recognition)	19.54 ± 1.05	19.38 ± 1.18	18.64 ± 1.73^**#^	*F* = 6.833; P^**^ = 0.003, *P*^#^ = 0.011
Delayed memory (Story Recall)	7.52 ± 3.74	7.14 ± 3.70	4.46 ± 2.98^**##^	*F* = 13.17; P^**^<0.001, *P*^##^<0.001
Delayed memory (Figure Recall)	14.46 ± 4.71	13.59 ± 4.26	10.70 ± 4.97^**#^	*F* = 9.908; P^**^<0.001, *P*^#^ = 0.003

HC: healthy control; MDD: major depressive disorder; BD: bipolar disorder; BMI: Body Mass Index; HAMD: Hamilton Depression Scale; HAMA: Hamilton Anxiety Scale; RBANS: Repeatable Battery for the Assessment of Neuropsychological Status; *p < 0.05, **p < 0.01 compared to HC group; #*p* < 0.05, ##*p* < 0.01 compared to MDD group.

**Table 2:** Comparison of MRI data among HC, MDD and BD.

	HC	MDD	BD
Volume (cm^3^)			
Hippocampus (Left)	3.69 ± 0.36	3.69 ± 0.36	3.70 ± 0.31
Hippocampus (Right)	3.62 ± 0.35	3.88 ± 0.34^**^	3.61 ± 0.30^##^
Parahippocampal gyrus (Left)	3.46 ± 0.31	3.37 ± 0.31	3.48 ± 0.32
Parahippocampal gyrus (Right)	4.04 ± 0.42	3.89 ± 0.39	4.00 ± 0.39
FALFF (a.u.)			
Hippocampus (Left)	−0.64 ± 0.17	−0.66 ± 0.15	−0.57 ± 0.16^*##^
Hippocampus (Right)	−0.63 ± 0.16	−0.61 ± 0.18	−0.50 ± 0.17^**##^
Parahippocampal gyrus (Left)	−0.52 ± 0.25	−0.47 ± 0.23	−0.45 ± 0.24
Parahippocampal gyrus (Right)	−0.51 ± 0.21	−0.47 ± 0.21	−0.42 ± 0.22
ReHo (a.u.)			
Hippocampus (Left)	−0.81 ± 0.19	−0.88 ± 0.14	−0.75 ± 0.15^##^
Hippocampus (Right)	−0.88 ± 0.17	−0.85 ± 0.15	−0.75 ± 0.16^**##^
Parahippocampal gyrus (Left)	−0.56 ± 0.18	−0.61 ± 0.18	−0.50 ± 0.20^##^
Parahippocampal gyrus (Right)	−0.61 ± 0.17	−0.66 ± 0.20	−0.54 ± 0.18^##^

HC: healthy control; MDD: major depressive disorder; BD: bipolar disorder; FALFF: fractional amplitude of low frequency fluctuation; ReHo: regional homogeneity; The fALFF and ReHo values were measured in normalized unit arbitrary unit (a.u.). *p < 0.05, **p < 0.01 compared to HC group; #p < 0.05, ##p < 0.01 compared to MDD group.

### Hippocampus and Parahippocampal gyrus findings

In terms of volume, the right hippocampus volume was increased in the MDD group (*p* < 0.001), while in the BD group, it was decreased (*p* < 0.01) compared to the MDD group. However, there was no significant difference in right hippocampus volume between the HC and BD groups. In terms of functional measures, the BD group exhibited increased fALFF values in the hippocampus (left and right) compared to both the HC and MDD groups (*p* < 0.01). In addition, the BD group showed significantly increased regional homogeneity (ReHo) values in the hippocampus (left and right) and parahippocampal gyrus (left and right) compared to the HC group (*p* < 0.01). Moreover, the BD group demonstrated significantly higher ReHo values in the right hippocampus compared to the MDD group (*p* < 0.01).

### Pearson correlation analysis

The results of Pearson correlation analysis revealed several significant associations (Table 3). Specifically, the ReHo values in the right hippocampus were positively correlated with HAMD scores (r = 0.32, *p* = 0.046) and HAMA scores (r = 0.27, p = 0.04). However, there were no significant correlations found between the volume, fALFF, and ReHo of the hippocampus (left and right) and parahippocampal gyrus (left and right) with total RBANS scores.

**Table 3:** Pearson correlation analysis of the indicators in the hippocampus and parahippocampal gyrus with the severity of BD and cognitive function.

		Volume				FALFF				ReHo			
		Hippocampus (Left)	Hippocampus (Right)	Parahippocampal gyrus (Left)	Parahippocampal gyrus (Right)	Hippocampus (Left)	Hippocampus (Right)	Parahippocampal gyrus (Left)	Parahippocampal gyrus (Right)	Hippocampus (Left)	Hippocampus (Right)	Parahippocampal gyrus (Left)	Parahippocampal gyrus (Right)
HAMD	r	−0.03	0.02	−0.002	0.04	0.06	0.02	0.03	0.04	−0.08	0.32	−0.09	−0.01
	P	0.84	0.87	0.99	0.76	0.63	0.88	0.85	0.78	0.53	0.046^*^	0.49	0.92
HAMA	r	−0.04	−0.03	−0.02	0.005	0.08	0.004	0.1	0.06	−0.08	0.27	−0.13	0.008
	P	0.78	0.85	0.9	0.97	0.54	0.98	0.44	0.66	0.56	0.04^*^	0.34	0.95
RBNAS	r	−0.03	−0.004	−0.17	−0.08	−0.14	0.09	0.02	0.06	−0.19	−0.01	0.08	−0.005
	P	0.8	0.98	0.19	0.53	0.27	0.48	0.88	0.66	0.14	0.93	0.54	0.97
Immediate memory (Learning)	r	−0.1	−0.009	−0.25	−0.17	−0.11	0.09	0.12	0.19	−0.12	0.03	0.32	0.08
	P	0.45	0.95	0.037^*^	0.2	0.41	0.51	0.34	0.15	0.36	0.8	0.04^*^	0.53
Immediate memory (Story Memory)	r	0.02	0.009	0.04	0.02	−0.04	0.27	0.02	0.018	−0.22	0.19	−0.04	−0.03
	P	0.86	0.95	0.74	0.86	0.77	0.035^*^	0.87	0.89	0.086	0.15	0.74	0.81
Visuospatial Construction	r	0.19	0.18	0.19	0.29	−0.22	−0.1	0.003	−0.08	−0.18	−0.02	−0.02	0.05
	P	0.14	0.17	0.14	0.04^*^	0.09	0.44	0.98	0.52	0.16	0.85	0.89	0.68
Language	r	−0.11	−0.12	−0.29	−0.18	−0.03	0.17	0.06	0.17	−0.04	0.01	−0.01	−0.06
	P	0.42	0.36	0.02^*^	0.17	0.8	0.19	0.65	0.19	0.74	0.93	0.92	0.65
Attention (Digit Span)	r	0.17	0.16	0.1	0.16	−0.16	−0.04	−0.06	−0.1	−0.17	0.03	0.03	−0.05
	P	0.2	0.2	0.46	0.23	0.22	0.75	0.63	0.47	0.2	0.84	0.79	0.71
Attention (Coding)	r	−0.08	−0.05	−0.32	−0.13	−0.11	−0.03	−0.07	−0.004	−0.09	−0.12	0.09	0.01
	P	0.57	0.68	0.03^*^	0.31	0.38	0.81	0.6	0.97	0.5	0.36	0.47	0.92
Delayed memory (List Recall)	r	0.03	−0.03	−0.07	−0.06	−0.18	0.05	0.14	0.08	−0.34	0.01	0.01	−0.09
	P	0.82	0.84	0.6	0.65	0.18	0.69	0.27	0.53	0.03^*^	0.91	0.91	0.5
Delayed memory (List Recognition)	r	−0.11	−0.08	−0.14	−0.07	0.05	0.11	0.12	0.13	−0.41	−0.07	0.002	−0.05
	P	0.39	0.55	0.28	0.62	0.7	0.4	0.36	0.32	0.02^*^	0.58	0.99	0.72
Delayed memory (Story Recall)	r	−0.04	−0.02	−0.11	−0.09	−0.04	0.27	−0.01	0.03	−0.09	0.15	−0.01	−0.09
	P	0.79	0.9	0.38	0.51	0.78	0.03^*^	0.93	0.83	0.5	0.24	0.93	0.5
Delayed memory (Figure Recall)	r	0.02	0.08	−0.03	0.02	−0.12	0.05	0.007	−0.02	−0.17	−0.05	0.04	0.02
	P	0.88	0.54	0.81	0.86	0.35	0.73	0.96	0.88	0.2	0.71	0.74	0.89

HAMD: Hamilton Depression Scale; HAMA: Hamilton Anxiety Scale; RBANS: Repeatable Battery for the Assessment of Neuropsychological Status.

Moreover, the volume of the left parahippocampal gyrus exhibited negative correlations with immediate memory (learning) (r = −0.25, *p* = 0.037), language (r = −0.29, *p* = 0.02), and attention (coding) (*r* = −0.32, *p* = 0.03). On the other hand, the volume of the right parahippocampal gyrus showed a positive correlation with visuospatial construction (r = 0.29, *p* = 0.04).

In terms of functional measures, the fALFF value of the right hippocampus was positively correlated with immediate memory (story memory) (r = 0.27, *p* = 0.035) and delayed memory (story recall) (r = 0.27, *p* = 0.03). Additionally, the ReHo value of the left hippocampus was found to have negative correlations with delayed memory (list recall) (r = −0.34, *p* = 0.03) and delayed memory (list recognition) (*r* = −0.41, *p* = 0.02). Lastly, the ReHo value of the left parahippocampal gyrus exhibited a positive correlation with immediate memory (learning) (r = 0.32, *p* = 0.04). Further analysis with Bonferroni correction showed that there was no significance among the volume, fALFF, and ReHo of the hippocampus (left and right) and parahippocampal gyrus (left and right).

## Discussion

This study utilizes rsMRI technology and automatic segmentation tools to unveil insights into the gray matter volume and brain function indicators of the hippocampus and parahippocampal gyrus in individuals with Bipolar Disorder (BD). Additionally, we conducted correlation analyses with the severity of the disorder and cognitive function. Our findings underscore that cognitive impairment in Bipolar Depression is significantly more pronounced when compared to both Healthy Controls (HC) and Major Depressive Disorder (MDD) patients. Moreover, we established a strong connection between specific functions of the hippocampus, parahippocampal gyrus, cognitive function, and disease severity.

Cognitive dysfunction has consistently emerged as a prominent feature in both MDD and BD (24). This impairment is intricately linked to overall functional outcomes and plays a crucial role in disease prognosis (25). Previous reports have indicated that BD patients exhibit more severe cognitive deficits compared to MDD patients (26). Our study confirms these observations, demonstrating that BD patients experience more pronounced cognitive dysfunction than HC and MDD groups. Specifically, the BD group displayed significant disparities in immediate memory, attention, and delayed memory when compared to the HC group, aligning with earlier research (27). It’s important to note that there were no significant cognitive impairments detected in any of the MDD groups, potentially attributed to the relatively small sample size of MDD patients.

MRI studies in the context of psychiatric disorders have consistently reported abnormal hippocampal volumes, influenced by various factors (28). Some studies suggest that structural changes in the hippocampus are state-dependent, occurring during acute phases of MDD and returning to normal after remission (29). Conversely, exercise has been associated with increased hippocampal volume (30). Our study reveals a significant increase in the right hippocampal volume of MDD patients, while no significant differences were observed in the BD group compared to the HC group. This suggests that factors such as age, medication, exercise, and others may exert influence on hippocampal volume (30).

Previous research has noted abnormal brain activity in BD patients, closely linked to their cognitive function, potentially serving as a means to differentiate BD from MDD patients (31). Significant differences were observed in brain regions encompassing the ventral and dorsolateral prefrontal cortex, insula, and putamen (32). However, there have been limited studies examining the global neural activity characteristics of the hippocampus and parahippocampal gyrus in BD and MDD patients using fALFF and ReHo values. In our study, we compared fALFF and ReHo, which provide insights into the strength and synchronization of local neural signals in the hippocampus and parahippocampal gyrus. Our results indicated that BD patients exhibited enhanced neural activity in the hippocampus (both left and right) compared to the HC and MDD groups. Furthermore, in terms of synchronization, both the hippocampus (left and right) and parahippocampal gyrus (left and right) showed elevated ReHo in BD patients when compared to the HC and MDD groups. These findings align with functional imaging studies that have highlighted abnormal brain activation in the hippocampus and parahippocampal gyrus during attention, emotional, and memory-related tasks. This consistency with neuropsychological findings, which reveal cognitive impairments during acute emotional episodes and significant declarative memory impairment during remission (33, 34), suggests that abnormal activity in the hippocampus and parahippocampal gyrus, as cognitive control regions, could potentially serve as biomarkers for distinguishing between BD and MDD.

In our study, we conducted Pearson correlation analyses between hippocampal and parahippocampal gyrus volumes, fALFF, ReHo, and cognitive function in BD patients. Interestingly, we found that the volumes of the hippocampus and parahippocampal gyrus showed no significant differences concerning HAMD, HAMA, and RBANS scores, contradicting some previous findings (14, 35). This discrepancy may be attributed to the specific characteristics of our study participants, who exhibited a relatively short course of BD with no functional abnormalities during the MRI process (36, 37). Regarding cognitive processes, previous research has emphasized the centrality of the hippocampus (38, 39). Our results supported this notion by revealing the involvement of the hippocampus in memory and attention functions. Additionally, we found a positive correlation between fALFF values in the hippocampus and parahippocampal gyrus and immediate and delayed memory, consistent with previous studies (40, 41). Furthermore, our study explored the less-studied Pearson correlation between hippocampal ReHo values and depressive scores, revealing a positive correlation between hippocampal ReHo values and HAMD and HAMA scores.

Nonetheless, several limitations warrant consideration in our study. One limitation of the present study is the lack of assessment regarding potential protective factors through psychotherapy and counseling intervention. Although individuals often utilize these non-pharmacological treatments without prescription to prevent or alleviate symptoms at the onset of mental illness (42), such information was not collected or analyzed in our study. Consequently, the potential influence of these protective factors on the observed MRI abnormalities remains unknown. Future research should consider incorporating measures of psychotherapy and counseling intervention to provide a more comprehensive understanding of their potential impact on functional and structural MRI abnormalities in bipolar and unipolar depression. Secondly, the uncontrolled effects of medications, despite general alignment with prior research, remain a limitation. Although the patients were drug-naïve, they may have been prescribed medications or other medical conditions. Additionally, the MDD patients included in our study exhibited a younger onset age compared to BD patients, which represents an atypical feature of depressive episodes and is considered a risk factor for BD (43). Lastly, our relatively small sample size, while comparable to previous studies, may limit the generalizability of our findings (44). Future research should endeavor to combine clinical phenotypes and employ longitudinal methods to replicate our results and provide more conclusive evidence.

## Conclusion

In conclusion, our study reveals distinctive intrinsic activity patterns in the hippocampus and parahippocampal gyrus of BD patients when compared to MDD and HC patients. These patterns may signify different underlying pathophysiological mechanisms in BD. Changes in fALFF and ReHo observed in the hippocampus and parahippocampal gyrus between BD and MDD patients are strongly associated with cognitive functions. Furthermore, the notable abnormal spontaneous neural activity detected in these regions may serve as a potential neural basis for distinguishing between bipolar depression and unipolar depression. Consequently, abnormal intrinsic brain activity opens up a new avenue for future research, shedding light on neuroimaging-based biomarkers for differentiating bipolar depression from unipolar depression.

## Data availability statement

The original contributions presented in the study are included in the article/supplementary material, further inquiries can be directed to the corresponding authors.

## Ethics statement

The studies involving humans were approved by the ethics committee of the Third People's Hospital of Foshan, China. The studies were conducted in accordance with the local legislation and institutional requirements. The participants provided their written informed consent to participate in this study.

## Author contributions

SH: Data curation, Writing – original draft. XW: Data curation, Writing – original draft. ZL: Data curation, Methodology, Writing – original draft. CL: Formal analysis, Methodology, Writing – original draft. YH: Formal analysis, Methodology, Writing – original draft. JL: Data curation, Writing – review & editing. WH: Funding acquisition, Writing – review & editing.
